# CD161 serves as a prognostic biomarker and predicts immunotherapy response in head and neck squamous cell carcinoma

**DOI:** 10.3389/fimmu.2026.1855876

**Published:** 2026-08-07

**Authors:** Shuai Mei, Xiaoyan Qian, Haizhu Chen, Xue Zhao, Yuqi Li, Haiyan Wang, Yunxia Tao

**Affiliations:** 1 Department of Oncology, The Affiliated Hospital of Xuzhou Medical University, Xuzhou, Jiangsu, China; 2 Department of Oncology, Henan Provincial People’s Hospital, People’s Hospital of Zhengzhou University, People’s Hospital of Henan University, Zhengzhou, Henan, China; 3 Breast Tumor Center, Sun Yat-sen Memorial Hospital, Sun Yat-sen University, Guangdong Provincial Key Laboratory of Malignant Tumor Epigenetics and Gene Regulation, Guangzhou, Guangdong, China; 4 Department of Pathology, Suqian Affiliated Hospital of Xuzhou Medical University, Suqian, Jiangsu, China; 5 School of Laboratory Medicine, Bengbu Medical University, Bengbu, Anhui, China

**Keywords:** CD161, head and neck squamous cell carcinoma, immune checkpoint inhibitors, prognostic biomarker, tumor microenvironment

## Abstract

**Background:**

Despite advances in immune checkpoint inhibitors (ICIs), the prognosis of patients with head and neck squamous cell carcinoma (HNSCC) remains poor, and reliable biomarkers for predicting clinical outcomes are still lacking. CD161, a lectin-like receptor expressed on multiple T-cell subsets, has been reported to inhibit T-cell cytotoxicity; however, its prognostic and immunological significance in HNSCC remains unclear.

**Methods:**

We analyzed transcriptomic data from the HNSCC cohort in the TCGA database and four GEO datasets (GSE6631, GSE102349, GSE159067, and GSE103322) to evaluate CD161 expression, its prognostic significance, and its association with immune infiltration and functional pathways. Single-cell RNA sequencing data were used to identify CD161-expressing cell subsets. Differential expression was further validated by immunohistochemistry (IHC) in 20 paired HNSCC and adjacent normal tissues. The association between CD161 expression and immunotherapy response was additionally assessed in an independent cohort of 33 ICI-treated patients.

**Results:**

CD161 expression was significantly lower in HNSCC tissues than in normal tissues in the TCGA cohort and the GSE6631 dataset, which was further confirmed by IHC analysis. High CD161 expression was associated with a favorable prognosis, with longer overall survival (OS) and disease-specific survival (DSS), and remained an independent protective factor in multivariate analysis (OS: hazard ratio [HR] = 0.92, 95% confidence interval [CI]: 0.86–0.99, p = 0.019; DSS: HR = 0.86, 95% CI: 0.78–0.96, p = 0.008). CD161 expression correlated with infiltration of CD8^+^ T cells and regulatory T cells, as well as with multiple immune checkpoint molecules, including CD27, TIGIT, BTLA, and PD-1. Single-cell analysis further localized CD161 predominantly to CD4^+^ T cells. In the GSE159067 dataset, high CD161 expression was associated with longer progression-free survival and overall survival. In our ICI-treated cohort, high CD161 expression correlated with longer PFS and higher objective response rate.

**Conclusion:**

CD161 is downregulated in HNSCC. Higher CD161 expression is associated with favorable prognosis and an immune-inflamed tumor microenvironment, and may predict response to ICI-containing therapy. These findings suggest that CD161 has potential as a prognostic biomarker and may help characterize tumor–immune interactions in HNSCC.

## 1 Introduction

Head and neck squamous cell carcinoma (HNSCC) is one of the most common malignancies and ranks as the sixth most common cancer worldwide. Globally, more than 900,000 new cases of HNSCC and over 450,000 related deaths occur annually (1). Approximately 60%–70% of patients are initially diagnosed with locally advanced or metastatic disease and have poor survival outcomes (2, 3). Although the advent of immune checkpoint inhibitors (ICIs) has significantly improved survival in a subset of patients (4, 5), immunosuppression and resistance to ICIs remain major causes of treatment failure (6–8). Therefore, identifying novel biomarkers that can predict therapeutic response and prognosis in patients with HNSCC receiving ICIs is of considerable clinical importance.

CD161, encoded by the killer cell lectin-like receptor B1 (*KLRB1*) gene, is widely expressed on multiple immune cell subsets, including CD4^+^ T cells, CD8^+^ T cells, and regulatory T cells (Tregs). CD161 has been identified as an inhibitory receptor on glioma-infiltrating T cells through its interaction with C-type lectin domain family 2 member D (CLEC2D). Previous studies have shown that CD161 negatively regulates antitumor immunity by suppressing T-cell cytotoxicity and cytokine production (9). High CD161 expression has been associated with aggressive clinicopathological features and poor prognosis in glioma (10). In addition, CD161^+^CD8^+^ T cells have been reported to be enriched in chemotherapy-resistant tumors and are associated with unfavorable clinical outcomes. Blockade of CD161 may help overcome tumor immune evasion and resistance to anticancer therapies (11). In contrast, elevated CD161 expression has been associated with improved survival in several malignancies, including lung cancer, breast cancer, and endometrial cancer (12–14).

In the present study, we compared CD161 expression between HNSCC and normal tissues, analyzed its association with clinicopathological characteristics, and evaluated its prognostic value using data from The Cancer Genome Atlas (TCGA) and Gene Expression Omnibus (GEO) databases. We further explored the biological functions associated with CD161 and examined its relationship with immune cell infiltration. Using single-cell RNA sequencing (scRNA-seq) data, we assessed its distribution across different cell populations, with further characterization in T cell subsets. The potential value of CD161 in predicting outcomes following ICI therapy was additionally evaluated using publicly available datasets. We further validated the differential expression of CD161 in paired HNSCC tumor-normal tissues and evaluated its association with clinical outcomes in an independent ICI-treated HNSCC cohort from our center. Our findings suggest that CD161 may serve as a prognostic biomarker and may help characterize tumor-immune interactions in HNSCC.

## 2 Materials and methods

### 2.1 Patients and samples

We obtained the mRNA expression profiles and corresponding clinical information for HNSCC from The Cancer Genome Atlas (TCGA) and Gene Expression Omnibus (GEO) databases. The HNSCC cohort from TCGA included 520 tumor samples and 44 normal tissue samples. Detailed clinicopathological characteristics and CD161 expression data are summarized in **Supplementary Table 1**. Four publicly available HNSCC-related datasets were downloaded from the GEO database: GSE6631, GSE102349, and GSE159067 for external validation, and GSE103322 for single-cell analysis. To validate the differential expression of CD161, immunohistochemical (IHC) staining was performed in 20 paired HNSCC and adjacent non-tumor tissue samples from our center. In addition, an independent cohort of 33 HNSCC patients who received ICI-containing therapy between October 2019 and September 2024 was retrospectively enrolled to evaluate the association between CD161 expression and immunotherapy outcomes. Eligible patients had locally advanced or metastatic HNSCC, received at least two cycles of ICI-containing therapy, had measurable lesions for response evaluation, and had complete treatment and follow-up data. Tumor response was evaluated according to the Response Evaluation Criteria in Solid Tumors (RECIST) version 1.1. Objective response rate (ORR) was defined as the proportion of patients achieving complete response (CR) or partial response (PR). Disease control rate (DCR) was defined as the proportion of patients achieving CR, PR, or stable disease (SD). Progression-free survival (PFS) was measured from initiation of ICI therapy to disease progression or death from any cause. There was no overlap between the two cohorts. Baseline clinical characteristics of the immunotherapy cohort are presented in **Table 1**. This study was approved by the Ethics Committee of the Affiliated Hospital of Xuzhou Medical University (Approval No. XYFY2024-KL482-01) and was conducted in accordance with the Declaration of Helsinki.

**Table 1:** Baseline characteristics in patients stratified by median CD161 expression in the immunotherapy cohort from our center.

Characteristics	Total (n = 33)	Low expression group (n = 17)	High expression group (n = 16)	p
	n (%)	n (%)	n (%)	
Gender				0.656
Male	28 (84.8)	15 (88.2)	13 (81.3)	
Female	5 (15.2)	2 (11.8)	3 (18.8)	
Age				0.296
≥ 60 years	20 (60.6)	12 (70.6)	8 (50.0)	
< 60 years	13 (39.4)	5 (29.4)	8 (50.0)	
Smoking				0.259
Yes	10 (30.3)	7 (41.2)	3 (18.8)	
No	23 (69.7)	10 (58.8)	13 (81.3)	
ECOG PS				0.303
0	15 (45.5)	6 (35.3)	9 (56.3)	
1	18 (54.5)	11 (64.7)	7 (43.8)	
Tumor site				1.000
Nasopharynx	22 (66.7)	11 (64.7)	11 (68.8)	
Others	11 (33.3)	6 (35.3)	5 (31.3)	
Differentiation Grade				0.157
Undifferentiated/Low	21 (63.6)	13 (76.5)	8 (50.0)	
Moderate/High	12 (36.4)	4 (23.5)	8 (50.0)	
EBER				1.000
Positive	22 (66.7)	11 (64.7)	11 (68.8)	
Negative	11 (33.3)	6 (35.3)	5 (31.3)	
T staging				0.259
T_1-2_	9 (27.3)	3 (17.6)	6 (37.5)	
T_3-4_	24 (72.7)	14 (82.4)	10 (62.5)	
N staging				0.259
N_0-1_	9 (27.3)	3 (17.6)	6 (37.5)	
N_2-3_	24 (72.7)	14 (82.4)	10 (62.5)	
M staging				0.721
M_0_	22 (66.7)	12 (70.6)	10 (62.5)	
M_1_	11 (33.3)	5 (29.4)	6 (37.5)	
Treatment				0.481
Curative	12 (36.4)	5 (29.4)	7 (43.8)	
Non-curative	21 (63.6)	12 (70.6)	9 (56.3)	

ECOG PS, Eastern Cooperative Oncology Group performance status; EBER, Epstein-Barr virus-encoded small RNA.

Others: Including hypopharynx (10 cases) and oropharynx (1 case). Curative treatment details: 6 cases received concurrent chemoradiotherapy combined with immunotherapy; 4 cases received immunotherapy as induction therapy; 1 case received immunotherapy as consolidation therapy and 1 case received immunotherapy as induction and consolidation therapy.

### 2.2 Immunohistochemistry staining

IHC staining was performed on tissue samples obtained from a total of 53 HNSCC patients from our center and was conducted by Wuhan Servicebio Technology Co., Ltd. (Wuhan, China). Tissue sections were first deparaffinized in xylene and rehydrated through a graded ethanol series. Antigen retrieval was performed by heating the sections in citrate buffer (pH 6.0). After blocking endogenous peroxidase activity, the sections were incubated with 3% BSA at room temperature for 30 minutes to reduce non-specific binding. The sections were then incubated with the primary antibody against CD161 (Catalog No. 67537-1-Ig, Proteintech, Wuhan, China), followed by incubation with the corresponding secondary antibody (Servicebio, GB27301). Antigen–antibody complexes were visualized using 3,3′-diaminobenzidine (DAB) staining. Finally, the sections were counterstained with hematoxylin, rinsed with running water, dehydrated, and mounted for microscopic examination.

IHC images were captured using an optical microscope and analyzed using ImageJ software. The “Colour Deconvolution” plugin was used to separate the staining channels and isolate the DAB-positive signal (Color_1). The Color_1 images were thresholded (0–220) and converted into binary masks. Image restoration was performed using the “Fill Holes” and “Watershed” functions. Positive staining areas were quantified using the “Analyze Particles” function. For optical density (OD) analysis, the Color_2 channel was converted to an 8-bit image and calibrated to obtain uncalibrated OD values. A threshold range of 0–220 was applied to calculate the average optical density (AOD). For the immunotherapy cohort, the mean density of five randomly selected regions at 10×40 magnification was calculated for each sample, and the average value was used to represent the final expression level. For the paired tumor and adjacent normal tissue cohort, the mean density was calculated from two regions at 10×20 magnification and two regions at 10×40 magnification for each sample.

### 2.3 Prognostic value of CD161

We first compared CD161 expression between tumor and normal tissues in the TCGA cohort and validated the findings using the GSE6631 dataset and 20 paired tissue samples from our center. The associations between CD161 expression and clinical characteristics, including age (≤ 60 vs. > 60 years), gender, T stage, N stage, and overall tumor stage, were evaluated.

Patients in the TCGA cohort were divided into high- and low-CD161 expression groups according to the optimal cutoff value. Survival differences between the two groups were assessed in terms of overall survival (OS) and disease-specific survival (DSS). Univariate and multivariate Cox regression analyses were then performed to identify independent prognostic factors associated with OS and DSS, including gender, age, tumor grade, clinical stage, alcohol consumption history, HPV status, and CD161 expression.

Kaplan–Meier survival analysis was further performed using the GSE102349 dataset, which contains gene expression and survival data, to validate the prognostic significance of CD161. Furthermore, the GSE159067 dataset, which includes 102 patients with advanced HNSCC who received immunotherapy, was used to assess the potential role of CD161 in predicting immunotherapy outcomes. In addition, the association between CD161 expression and PFS was evaluated in our institutional immunotherapy cohort.

### 2.4 CD161 enrichment analysis

To explore the potential biological functions and signaling pathways associated with different levels of CD161 expression in HNSCC, patients in the TCGA cohort were stratified into CD161-high and CD161-low expression groups according to the median CD161 expression level. This grouping strategy was selected to provide balanced sample sizes and to facilitate the comparison of transcriptomic profiles associated with distinct CD161 expression states. Differentially expressed genes (DEGs) between the CD161-high and CD161-low groups were identified using the DESeq2 package in R. DEGs were defined as genes with an absolute log2 fold change > 2 and an adjusted p-value (false discovery rate, FDR) < 0.05, with the Benjamini–Hochberg method applied for multiple testing correction. A volcano plot highlighting the most significantly differentially expressed genes between the CD161-high and CD161-low groups is presented in **Supplementary Figure 1**.

Functional enrichment analyses, including Gene Ontology (GO) and Kyoto Encyclopedia of Genes and Genomes (KEGG) pathway analyses, were conducted using the clusterProfiler package. In addition, Gene Set Enrichment Analysis (GSEA) was performed using the MSigDB C5 collection (version 7.0). Genes were ranked according to their log2 fold changes, and enrichment analysis was performed between the high- and low-CD161 expression groups. Pathways with p < 0.05 were considered significantly enriched, and the top eight pathways were selected according to ascending p-values.

### 2.5 Correlation of CD161 expression with immune cell infiltration and immune checkpoint

Based on the enrichment analysis results, Spearman’s correlation analysis was performed to evaluate the relationship between CD161 expression and immune cell infiltration in HNSCC. Pearson correlation analysis was used to assess the associations between *KLRB1* (encoding CD161) expression and the expression levels of immune checkpoint-related genes, including CD137, VISTA, PD-L1, TIM3, CD27, HVEM, CTLA-4, ICOS, CD28, TIGIT, BTLA, OX40, GITR, and PD-1. Correlation results were visualized using heatmaps generated with the corrplot package.

### 2.6 scRNA-seq analysis

The scRNA-seq dataset GSE103322 was analyzed using the Seurat package in R. Cells with fewer than 200 detected genes or more than 20% mitochondrial gene content were excluded. After quality control, a total of 5,902 cells were retained for downstream analysis. Potential doublets were removed during preprocessing using standard quality control procedures. Batch effects were corrected using Seurat integration methods. Data normalization was performed following the standard Seurat workflow. Dimensionality reduction was performed using principal component analysis (PCA), and Uniform Manifold Approximation and Projection (UMAP) was applied for visualization. Cell clustering was conducted based on the first significant principal components, and marker genes were identified for each cluster for cell-type annotation. The expression of CD161 across different clusters was then analyzed to determine its cellular distribution in the tumor microenvironment.

### 2.7 Statistical analysis

Differential expression analyses of CD161 between tumor and normal tissues were performed using the Mann–Whitney U test for the TCGA cohort, the Wilcoxon signed-rank test for the paired institutional cohort, and the paired-sample t-test for the GSE6631 dataset. For the TCGA and GEO cohorts, optimal cutoff values for survival analyses were determined using the surv_cutpoint function in the R package survminer based on maximally selected rank statistics. The distribution of CD161 expression levels in the high- and low-expression subgroups is shown in **Supplementary Figure 2**. Survival curves were estimated using the Kaplan–Meier method and compared using the log-rank test. Univariate and multivariate analyses were performed using the Cox proportional hazards regression model. All statistical analyses were conducted using R software (version 4.0.3). A two-sided p < 0.05 was considered statistically significant.

## 3 Results

### 3.1 The decreased expression level of CD161 in HNSCC

**Figure 1:**
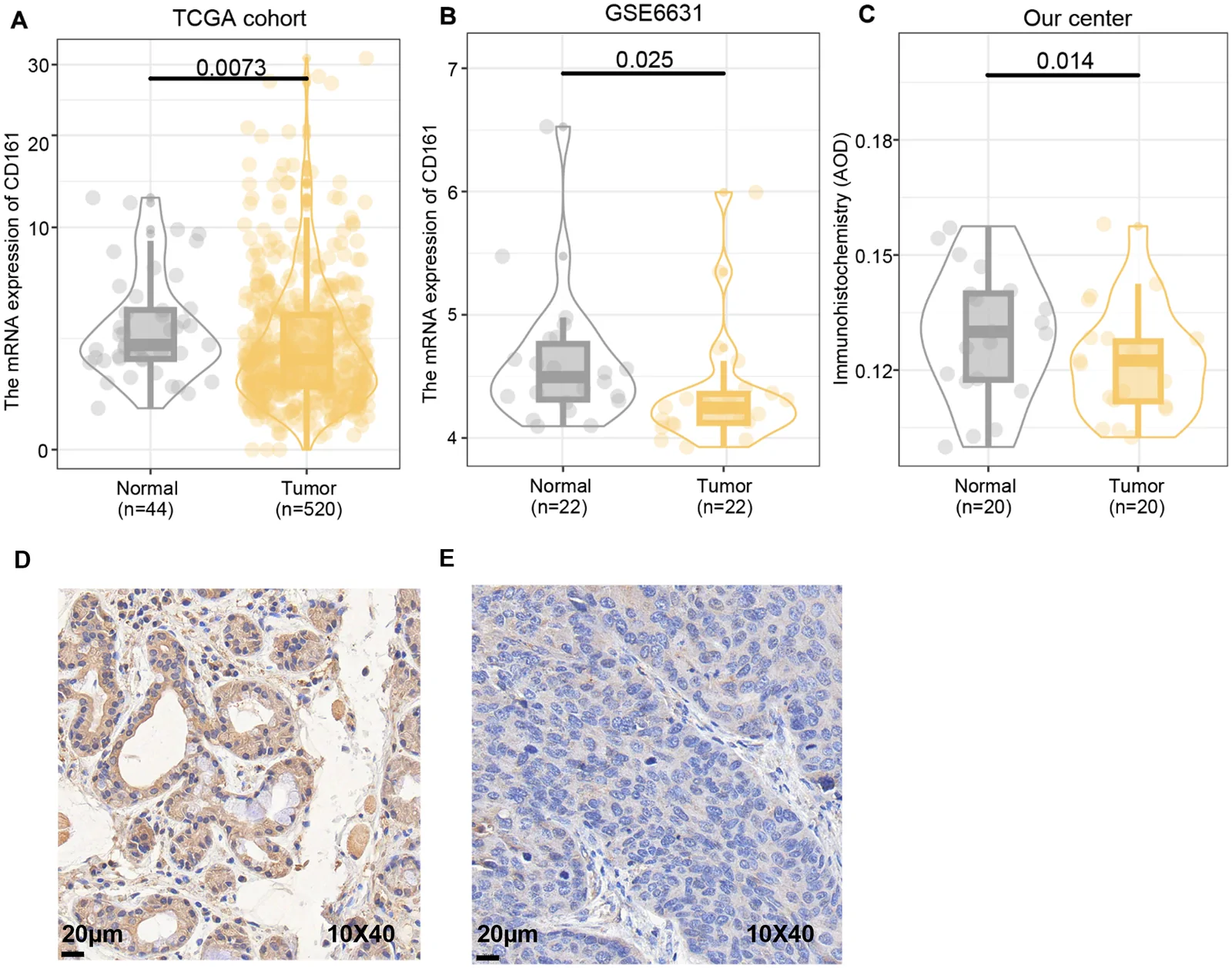
Differential expression of CD161 between tumor and normal tissues. **(A)** Comparison of CD161 mRNA levels between HNSCC tumor and normal tissues in TCGA cohort (Mann–Whitney U test, p = 0.0073); **(B)** Comparison of CD161 mRNA levels between HNSCC tumor and normal tissues in GSE6631 dataset (paired Student’s *t*-test, p = 0.025); **(C)** CD161 immunohistochemistry staining results between paired HNSCC tumor and adjacent normal tissues from our center (Wilcoxon signed-rank test, p = 0.014); **(D)** Representative immunohistochemical staining of CD161 in adjacent normal tissue from HNSCC patients (original magnification ×400); **(E)** Representative immunohistochemical staining of CD161 in HNSCC tumor tissue (original magnification ×400).

CD161 was significantly reduced in most tumor types compared to normal tissues, including bladder cancer, breast cancer, colon adenocarcinoma, and HNSCC (all p < 0.05; **Supplementary Figure 3**; **Figure 1A**). This decrease in HNSCC was validated using the GSE6631 dataset (p = 0.025, **Figure 1B**), and confirmed by IHC in 20 paired HNSCC and adjacent normal tissues from our center (p = 0.014, **Figure 1C**). Representative IHC images are shown in **Figures 1D**, **E**.

### 3.2 Association between CD161 expression and clinical characteristics

**Figure 2:**
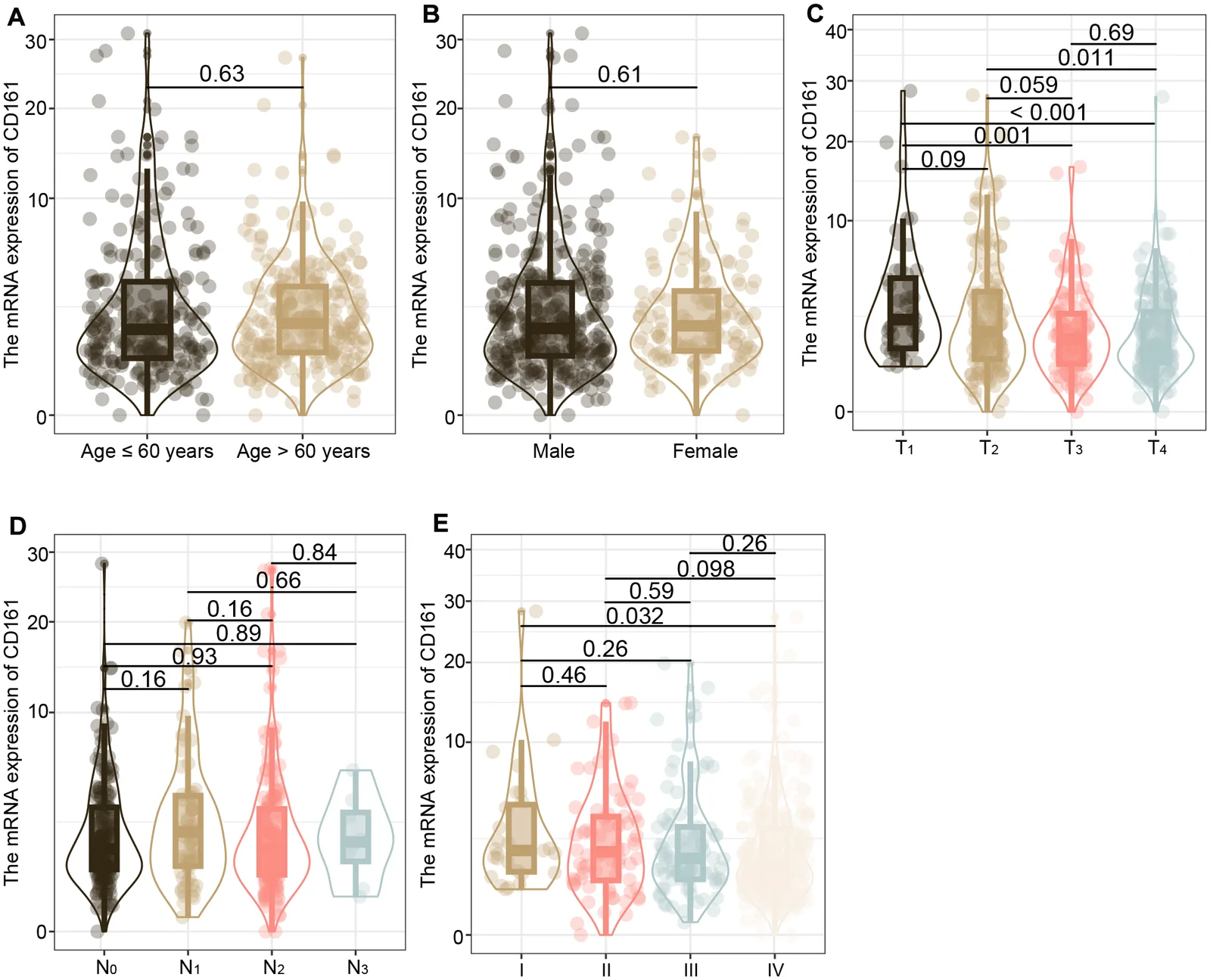
Association between CD161 expression and clinicopathological characteristics of HNSCC in the TCGA cohort. **(A)** Age-stratified (≤ 60years vs. >60 years) expression of CD161 (Mann–Whitney U test, p = 0.63); **(B)** Sex-stratified expression of CD161 (Mann–Whitney U test, p = 0.61); **(C)** CD161 expression based on T-staging; **(D)** CD161 expression based on N-staging; **(E)** CD161 expression based on cancer stages. Pairwise comparisons in panels C–E were performed using the Mann–Whitney U test, and the corresponding p values are indicated in the figure.

The associations between CD161 expression and clinicopathological characteristics in the TCGA cohort are shown in **Figure 2**. No significant differences in CD161 expression were observed according to age (≤ 60 years vs. > 60 years, **Figure 2A**) or gender (**Figure 2B**). For T stage, no statistically significant differences were observed between adjacent T stages (T_1_ vs. T_2_, T_2_ vs. T_3_, and T_3_ vs. T_4_; **Figure 2C**), but CD161 expression was significantly higher in T_1_ than in T_3_ and T_4_ tumors (both p < 0.05, **Figure 2C**). No significant differences were observed according to N stage (N_0_, N_1_, and N_2;_ **Figure 2D**) or tumor stage (**Figure 2E**), except between stage I and stage IV (p = 0.032, **Figure 2E**).

### 3.3 Prognostic significance of CD161 expression in HNSCC

**Figure 3:**
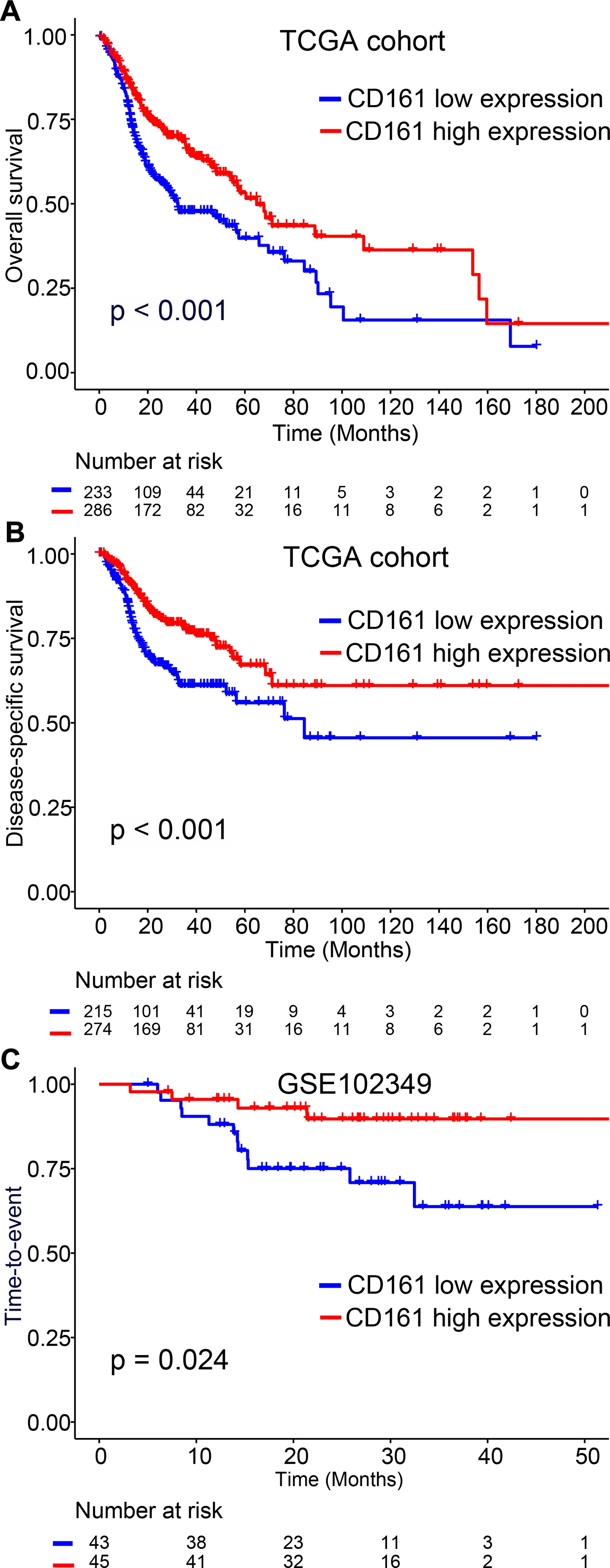
Kaplan–Meier survival analysis based on CD161 expression. **(A)** Overall survival in the TCGA cohort (log–rank test, p < 0.001); **(B)** Disease-specific survival in the TCGA cohort (log–rank test, p < 0.001); **(C)** Time-to-event in the GSE102349 dataset (log–rank test, p = 0.024).

Kaplan–Meier survival analysis of the TCGA cohor demonstrated that patients with high CD161 expression had significantly longer OS (p < 0.001, **Figure 3A**) and DSS (p < 0.001, **Figure 3B**) than those with low CD161 expression.

To further assess the independent prognostic significance of CD161, univariate and multivariate Cox regression analyses were performed for both OS and DSS in the TCGA cohort (**Table 2**). Multivariate analysis revealed that higher CD161 expression was independently associated with improved OS (hazard ratio [HR] = 0.92, 95% confidence interval [CI]: 0.86–0.99, p = 0.019) and DSS (HR = 0.86, 95% CI: 0.78–0.96, p = 0.008). Clinical stage was also identified as an independent prognostic factor for both OS (HR = 1.42, 95% CI: 1.18–1.71, p < 0.001) and DSS (HR = 1.45, 95% CI: 1.13–1.87, p = 0.003). Age was independently associated with OS only (HR = 1.02, 95% CI: 1.01–1.04, p = 0.005). In contrast, gender, tumor grade, alcohol consumption history, and HPV status were not independent predictors of either OS or DSS.

**Table 2:** Univariate and multivariate analyses for OS and DSS in the TCGA cohort.

Factors	OS				DSS			
	Univariate analysis		Multivariate analysis		Univariate analysis		Multivariate analysis	
	HR (95% CI)	p	HR (95% CI)	p	HR (95% CI)		HR (95% CI)	p
Gender (Female vs. Male)	1.35 (1.01-1.80)	0.040	1.24 (0.88-1.71)	0.214	0.99 (0.72-1.37)	0.953		
Age	1.02 (1.01-1.04)	0.001	1.02 (1.01-1.04)	0.005	1.01 (0.99-1.02)	0.217		
Grade	1.01 (0.83-1.23)	0.927			1.03 (0.84-1.28)	0.770		
Stage	1.41 (1.18-1.68)	<0.001	1.42 (1.18-1.71)	<0.001	1.54 (1.20-1.98)	0.001	1.45 (1.13-1.87)	0.003
Alcohol history (Yes vs. No)	1.07 (0.80-1.42)	0.653			1.21 (0.81-1.79)	0.354		
HPV status (positive vs. negative)	0.48 (0.29-0.79)	0.004	0.82 (0.46-1.47)	0.504	0.54 (0.30-0.98)	0.044	1.00 (0.49-2.03)	0.990
CD161	0.91 (0.86-0.96)	0.001	0.92 (0.86-0.99)	0.019	0.89 (0.83-0.96)	0.008	0.86 (0.78-0.96)	0.008

CI, confidence interval; DSS, disease-specific survival; HPV, human papilloma virus; HR, Hazard ratio; OS, overall survival.

The prognostic value of CD161 was further validated using the GSE102349 dataset. Patients with high CD161 expression exhibited significantly longer time-to-event (TTE) than those with low CD161 expression (p = 0.024, **Figure 3C**).

### 3.4 Functional enrichment analysis

Based on the median expression level of CD161, a total of 382 differentially expressed genes (DEGs) were identified in the TCGA cohort. To investigate the biological functions associated with CD161, Gene Ontology (GO), Kyoto Encyclopedia of Genes and Genomes (KEGG), and Gene Set Enrichment Analysis (GSEA) were performed using these DEGs.

**Figure 4:**
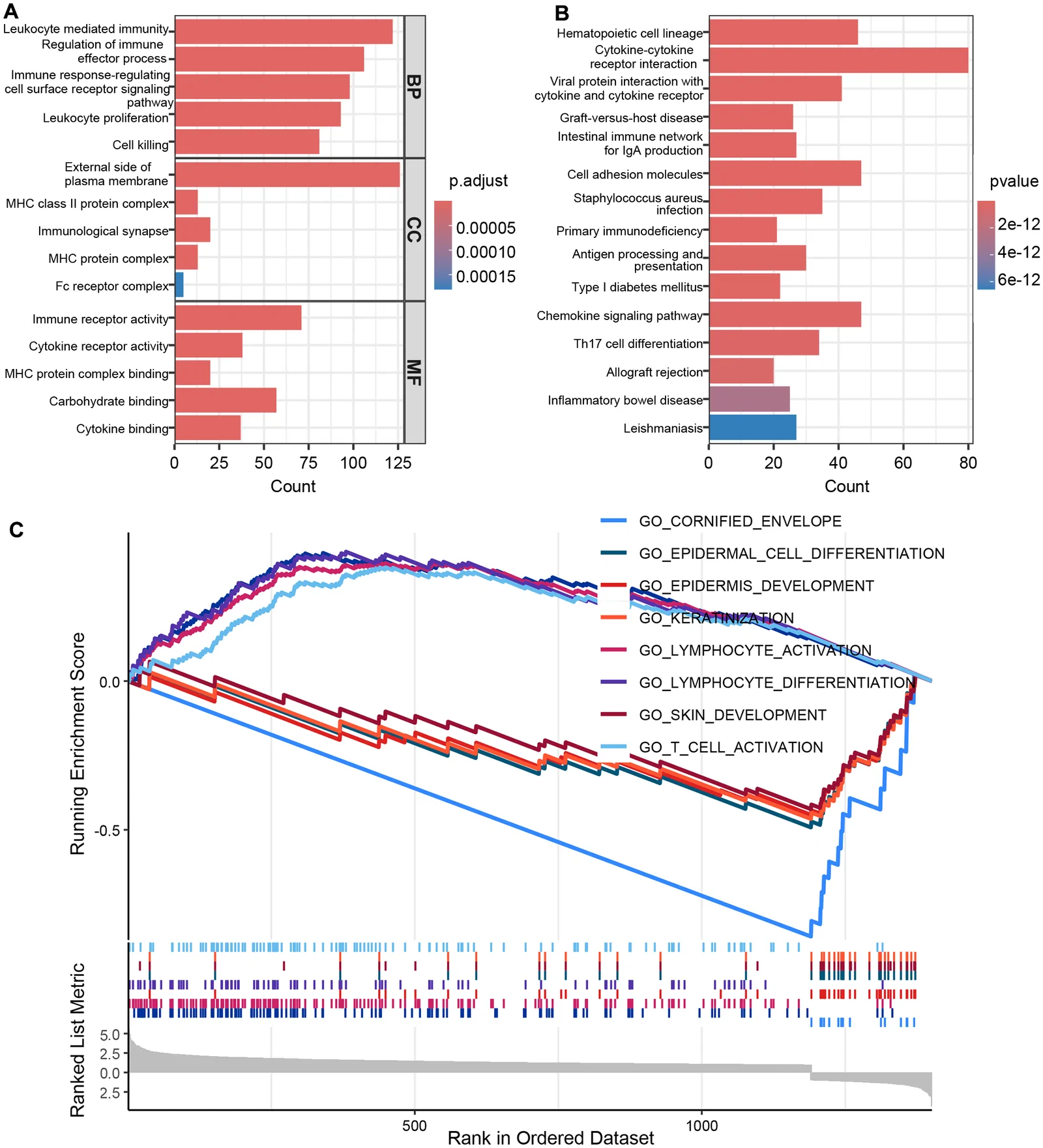
Functional enrichment analysis of CD161. **(A)** Gene Ontology analysis including biological processes (BP), cellular components (CC), and molecular function (MF); **(B)** Kyoto Encyclopedia of Genes and Genomes pathway enrichment analysis; **(C)** Gene Set Enrichment Analysis.

GO analysis indicated that CD161-associated genes were primarily enriched on the external side of the plasma membrane. Immune receptor activity was identified as the predominant molecular function. The most significantly enriched biological processes included leukocyte-mediated immunity and regulation of immune effector process. (**Figure 4A**).

KEGG pathway analysis revealed that cytokine–cytokine receptor interaction was the most significantly enriched signaling pathway associated with CD161 (**Figure 4B**). GSEA demonstrated that KLRB1 expression was positively associated with immune-related pathways, including lymphocyte activation and T-cell activation, whereas pathways related to epidermal cell differentiation and development were negatively enriched in HNSCC (**Figure 4C**).

### 3.5 Association between CD161 expression and immune cell infiltration

**Figure 5:**
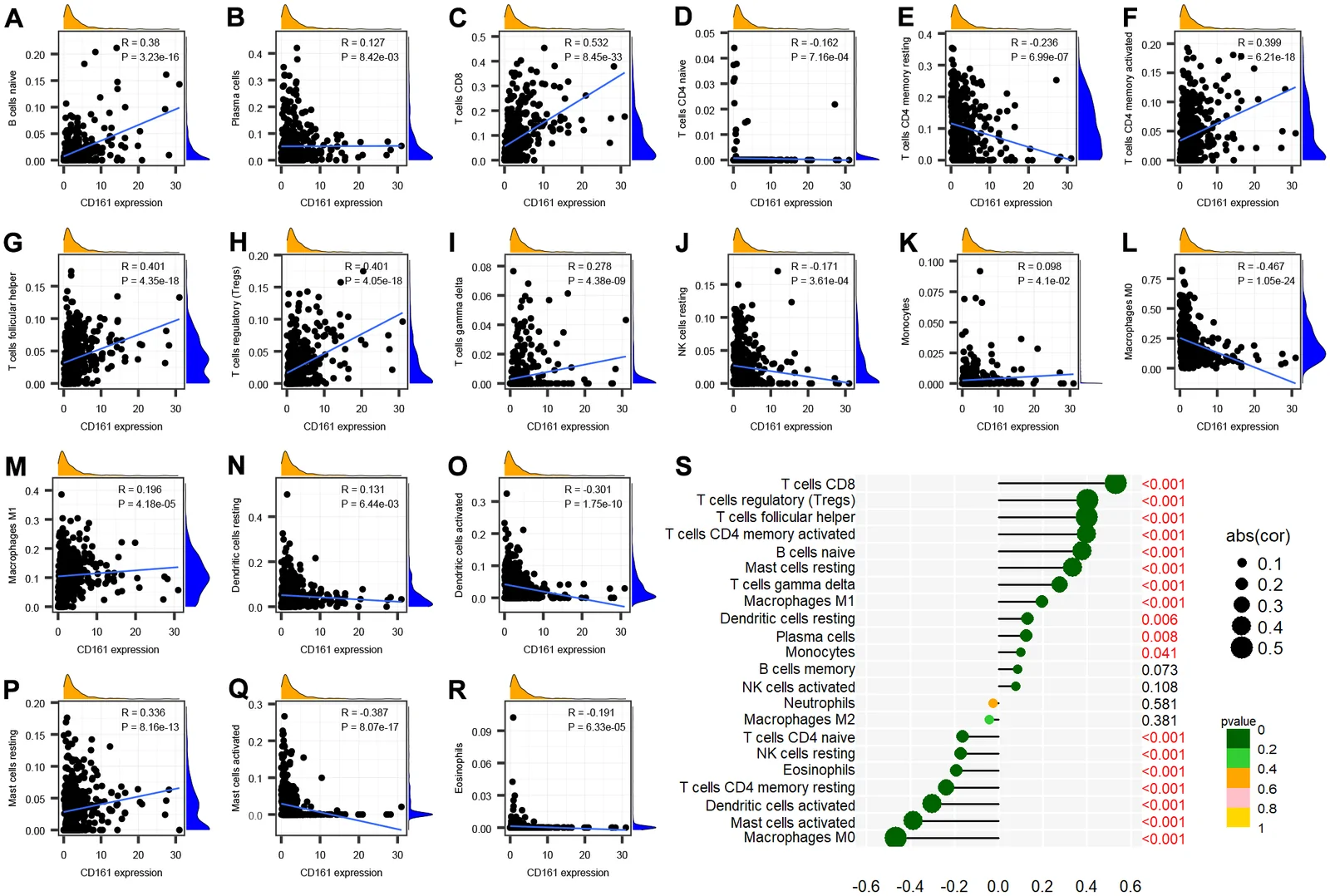
Association between CD161 expression and immune cell infiltration. **(A–R)** Spearman correlation analysis between CD161 expression and immune cell subtypes; **(S)** Summary lollipop plot of correlation coefficients across all immune cell types.

Spearman’s correlation analysis was performed to evaluate the relationship between CD161 expression and immune cell infiltration in the TCGA cohort. The results are presented in **Figures 5A–S**. CD161 expression was positively correlated with naïve B cells (R = 0.380, p < 0.001), plasma cells (R = 0.127, p = 0.008), CD8^+^ T cells (R = 0.532, p < 0.001), activated memory CD4^+^ T cells (R = 0.399, p < 0.001), T follicular helper (TFH) cells (R = 0.401, p < 0.001), Treg cells (R = 0.401, p < 0.001), γδ T cells (R = 0.278, p < 0.001), monocytes (R = 0.098, p = 0.041), M1 macrophages (R = 0.196, p < 0.001), resting dendritic cells (R = 0.131, p = 0.006), and resting mast cells (R = 0.336, p < 0.001). Conversely, CD161 expression was negatively correlated with naïve CD4^+^ T cells (R = −0.162, p < 0.001), resting memory CD4^+^ T cells (R = −0.236, p < 0.001), resting NK cells (R = −0.171, p < 0.001), M0 macrophages (R = −0.467, p < 0.001), activated dendritic cells (R = −0.301, p < 0.001), activated mast cells (R = −0.387, p < 0.001), and eosinophils (R = −0.191, p < 0.001).

**Figure 6:**
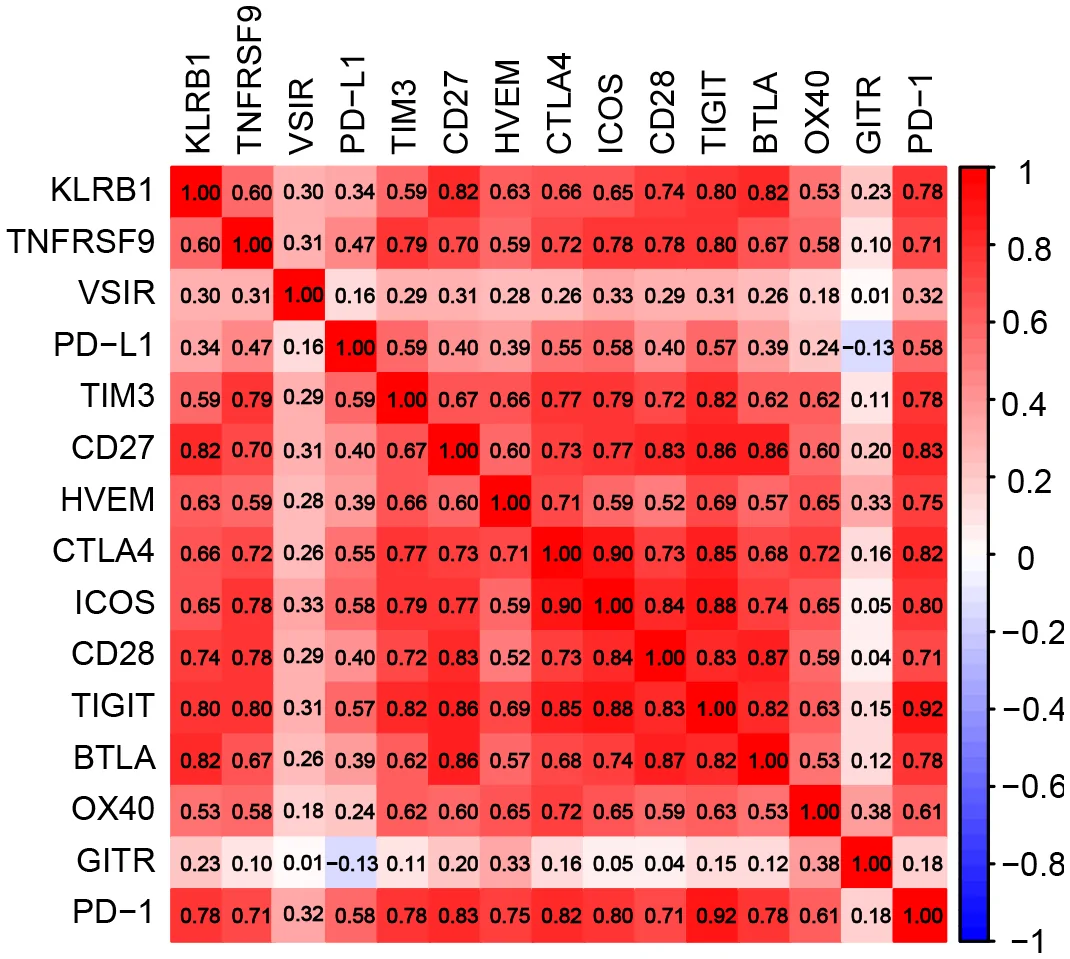
Pearson correlation analysis of CD161 (encoded by *KLRB1*) and immune checkpoints.

We further explored the association between CD161 and immune checkpoint molecules, including CD137, VISTA, PD-L1, TIM3, CD27, HVEM, CTLA-4, ICOS, CD28, TIGIT, BTLA, OX40, GITR, and PD-1. As shown in **Figure 6**, CD161 showed strong positive correlations with multiple immune checkpoints, most notably CD27 (R = 0.82), BTLA (R = 0.82), TIGIT (R = 0.80), and PD-1 (R = 0.78).

### 3.6 CD161 expression primarily in CD4^+^ T cells

To investigate the cellular distribution of CD161 in HNSCC, single-cell RNA sequencing analysis was performed using the GSE103322 dataset with Seurat package. We identified 20 distinct cell clusters (**Supplementary Figure 4**).

**Figure 7:**
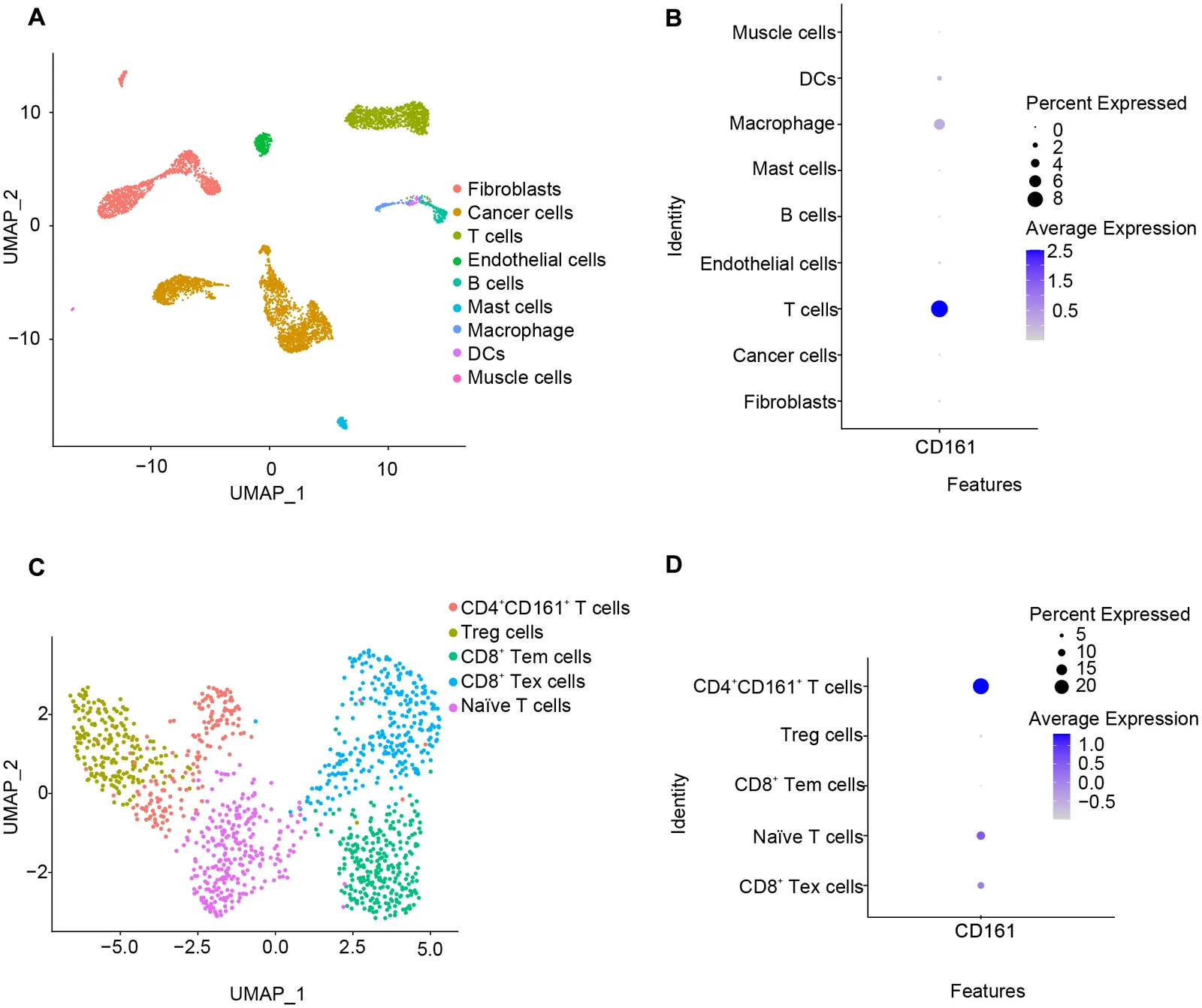
Single-cell analysis of CD161 expression. **(A)** Cell cluster map annotated by cell types; **(B)** Expression levels of CD161 across cell clusters; **(C)** T cell subclustering analysis; **(D)** Distribution of CD161 in T cell subsets.

Based on established marker genes, nine major cell populations were annotated, including fibroblasts (THY1, FAP, COL1A2, COL3A1, DCN, and COL6A1; clusters 0, 5, and 11), cancer cells (CDH1, EPCAM, and KRT8; clusters 1, 3, 4, 7, 8, 13, and 17), T cells (CD3E, CD3G, and CD3D; clusters 2, 6, and 10), endothelial cells (VWF, FLI1, and ERG; cluster 9), B cells (CD19, CD79A, and BLNK; cluster 12), mast cells (KIT, CMA1, MS4A2, TPSAB1, and TPSB2; cluster 14), macrophages (CD163, CLEC10A, and CSF1R; cluster 15), dendritic cells (CD86, CD80, and CCR7; cluster 18), and muscle cells (ACTN2, MYL2, and MYH2; cluster 19) (**Figure 7A**; **Supplementary Figure 5**).

CD161 expression was predominantly observed in T cell populations (**Figure 7B**). Further subclustering of T cells (**Supplementary Figures 6**, **7**) identified naïve T cells, regulatory T cells, CD8^+^ effector memory T cells, and CD8^+^ exhausted T cells (**Figure 7C**).Notably, a CD4^+^ T cell population with high CD161 expression was identified, which co-expressed markers associated with cytotoxicity (GZMB) and T cell activation/exhaustion (SELL, PD-1, and CTLA-4), suggesting a transitional or activated immune state rather than a clearly defined lineage subset (**Figure 7D**; **Supplementary Figure 7**).

### 3.7 The relationship between CD161 and prognosis of patients with HNSCC receiving immunotherapy

Based on the results of single-cell and enrichment analyses, CD161 appeared to be closely associated with immune-related biological processes. We therefore investigated whether CD161 expression was associated with clinical outcomes in HNSCC patients receiving immunotherapy.

**Figure 8:**
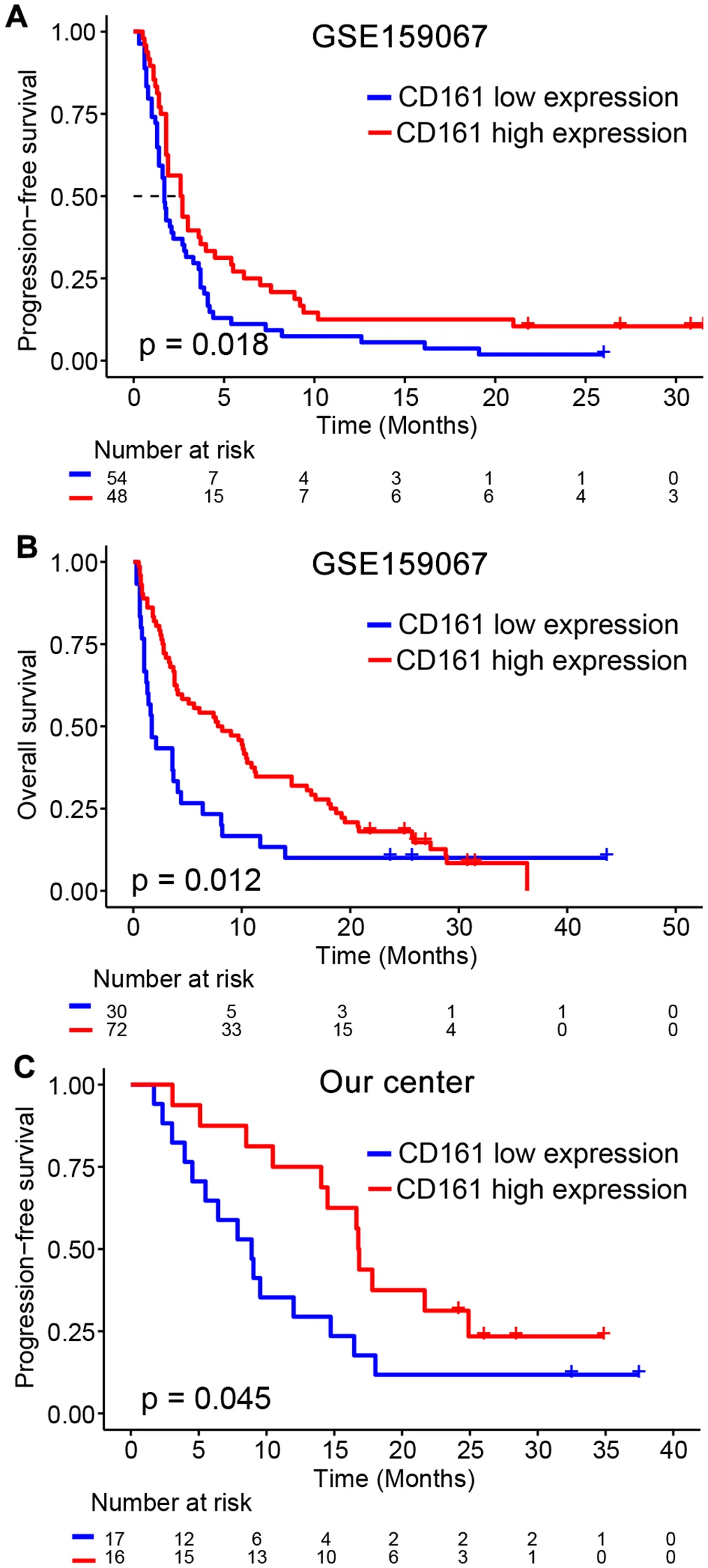
Kaplan–Meier survival analysis based on CD161 expression for HNSCC receiving immunotherapy. **(A)** Progression-free survival in the GSE159067 dataset (log-rank test, p = 0.018); **(B)** Overall survival in the GSE159067 dataset (log-rank test, p = 0.012); **(C)** Progression-free survival in our center (log-rank test, p = 0.045).

Analysis of the GSE159067 dataset demonstrated that patients with high CD161 expression had significantly longer PFS (p = 0.018, **Figure 8A**) and OS (p = 0.012, **Figure 8B**) than those with low CD161 expression.

The prognostic value of CD161 was further evaluated in our institutional immunotherapy cohort. A total of 33 patients were included, all of whom received anti–PD-1 inhibitors combined with chemotherapy. The cohort was predominantly male (84.8%), with 39.4% younger than 60 years, and 33.3% had distant metastases. Curative-intent treatment was administered to 12 patients (36.4%), whereas the remaining 21 (63.6%) received palliative-intent treatment. Among the 12 patients receiving curative-intent treatment, 6 received concurrent chemoradiotherapy combined with immunotherapy, whereas the other 6 received immunotherapy as induction (n = 4), consolidation (n = 1), or both induction and consolidation (n = 1) alongside chemoradiotherapy. Across the entire cohort, chemotherapy regimens included TP (taxane plus platinum, n = 7), GP (gemcitabine plus platinum, n = 18), and FP (fluorouracil plus platinum, n = 8). Detailed baseline characteristics are summarized in **Table 1**.

The median follow-up duration was 14.0 months (range, 8.9–17.8 months). Patients were divided into high- and low-CD161 expression groups according to the median AOD value. No significant differences in baseline characteristics were observed between the two groups (**Table 1**). In the high-CD161 group, the ORR was significantly higher than that in the low-CD161 group (75.0% vs. 35.3%, p = 0.037). The disease control rate (DCR) was numerically higher in the high-CD161 group (87.5% vs. 76.5%), although the difference did not reach statistical significance (p = 0.656). Detailed treatment response data are summarized in **Table 3**. Consistent with the findings from the public dataset, patients with low CD161 expression had significantly shorter PFS than those with high CD161 expression (8.9 months [95% CI, 5.5–16.5] vs. 16.8 months [95% CI, 14.0–NR]; p = 0.045, **Figure 8C**).

**Table 3:** Treatment response stratified by median CD161 expression in the immunotherapy cohort from our center.

Response	Total (n = 33)	Low expression group (n = 17)	High expression group (n = 16)	p
	n (%)	n (%)	n (%)	
CR				
No	25 (75.76)	14 (82.35)	11 (68.75)	
Yes	8 (24.24)	3 (17.65)	5 (31.25)	
PR				
No	23 (69.70)	14 (82.35)	9 (56.25)	
Yes	10 (30.30)	3 (17.65)	7 (43.75)	
SD				
No	24 (72.73)	10 (58.82)	14 (87.50)	
Yes	9 (27.27)	7 (41.18)	2 (12.50)	
PD				
No	27 (81.82)	13 (76.47)	14 (87.50)	
Yes	6 (18.18)	4 (23.53)	2 (12.50)	
ORR				0.037
No	15 (45.45)	11 (64.71)	4 (25.00)	
Yes	18 (54.55)	6 (35.29)	12 (75.00)	
DCR				0.656
No	6 (18.18)	4 (23.53)	2 (12.50)	
Yes	27 (81.82)	13 (76.47)	14 (87.50)	

CR, complete response; DCR, disease control rate; ORR, objective response rate; PD, progressive disease; PR, partial response; SD, stable disease.

## 4 Discussion

CD161 expressed on immune cells, including tumor-infiltrating CD4^+^ and CD8^+^ T cells as well as natural killer (NK) cells. It plays a key role in regulating antitumor immune responses (9, 15, 16). Emerging evidence suggests that CD161 signaling is highly context-dependent, with either inhibitory or activating effects on tumor progression depending on the cancer type, immune landscape, and the expression of its ligand CLEC2D (17–19). However, the functional role of CD161 in HNSCC remains largely undefined. Because immunosuppression and therapeutic resistance are major challenges in HNSCC, profiling CD161^+^ T cells in the tumor microenvironment could reveal novel prognostic biomarkers and therapeutic targets. To our knowledge, this study provides the first systematic evaluation of CD161 expression, clinical significance, and immunological implications in HNSCC.

In line with prior pan-cancer analysis (17), our data indicated that high CD161 expression was associated with better clinical outcomes in HNSCC. Notably, CD161 levels were significantly lower in HNSCC tumors than in the corresponding normal tissues, a finding validated in TCGA, GEO, and our institutional samples. Moreover, CD161 expression tends to be lower in more advanced T stage. These observations are consistent with reports that elevated CD161 tends to occur in early T stage which correlates with abundant tumor-infiltrating lymphocytes and improved prognosis (20, 21). Given that CD161 is primarily expressed on cytotoxic effector cells (NK cells and CD4^+^/CD8^+^ T cells), its reduction in advanced tumors may reflect enhanced immune evasion and weakened antitumor immunity (21).

Our analysis showed that high CD161 expression was significantly associated with improved OS and DSS in HNSCC patients. Importantly, CD161 expression remained an independent prognostic factor for OS and DSS in multivariate Cox models, underscoring its robust prognostic value. However, several clinically relevant variables, including smoking status and anatomical subsite, were not incorporated into the multivariate analyses because these data were unavailable or incomplete for a substantial proportion of patients in the TCGA cohort. Inclusion of these variables would have substantially reduced the number of evaluable cases and potentially introduced selection bias. Therefore, future studies with more comprehensive clinical annotations are warranted to further validate the independent prognostic significance of CD161 after adjustment for these established risk factors. Mechanistically, CD161 has been reported to function as a co-stimulatory receptor on T cells (22). Engagement of CD161 by its ligand LLT1 (CLEC2D) expressed on antigen-presenting cells or tumor cells triggers downstream signaling (such as the PLCγ PKC–ERK signaling pathway), which enhances T cell proliferation, effector cytokine production (IFN-γ and TNF-α), and cytotoxic activity (22). These mechanisms could underlie the improved clinical outcomes observed in HNSCC patients with elevated CD161 expression.

Our integrative transcriptomic analysis further clarified the immune context of CD161 in HNSCC. Functional enrichment analyses (GO, KEGG, and GSEA) revealed that CD161 was predominantly associated with pathways related to T cell activation, cytokine-receptor signaling, and immune cell-mediated responses (23, 24). Intriguingly, GSEA revealed that tumors with high CD161 expression were significantly negatively enriched for keratinocyte differentiation pathways, indicating a transition from an epithelial-like phenotype toward an immune-dominant microenvironment. This pattern is consistent with prior studies that showed that immune-active tumors are often characterized by suppression of epithelial differentiation programs and enhanced immune signaling (25). This immune-dominant shift could contribute to the favorable prognosis seen in patients with high CD161 levels.

At the single-cell level, CD161 was mainly expressed by CD4^+^ T cells, with comparatively lower expression in CD8^+^ T cell subsets. This distribution is functionally significant. In HPV-positive oropharyngeal squamous cell carcinoma, for example, high infiltration of CD4^+^CD161^+^ Tem cells correlated with better DSS, whereas CD8^+^CD161^+^ T cell infiltration does not have the same prognostic impact (26). The prognostic benefit associated with CD4^+^CD161^+^ Tem infiltration may be mechanistically linked to their enhanced functional capabilities. These cells exhibit heightened TCR signaling and reactivity, particularly under suboptimal antigen conditions. This is driven by their specific overexpression of the transcription factor SOX4, which transactivates CD3ϵ and amplifies TCR signaling. Additionally, they show increased responsiveness to innate cytokines. Furthermore, unlike classical inhibitory checkpoints, CD161 expression decreases upon T cell activation and is potently downregulated by TGFβ1, consistent with a highly responsive effector phenotype rather than an exhausted state (26). In the TCGA cohort, CD161 expression is associated with a strongly immune-inflamed microenvironment. In particular, CD161 levels correlate positively with multiple effector T-cell subsets, including CD8+ T cells, Tfh cells, and activated memory CD4+ T cells. Notably, CD161 expression was also positively correlated with Treg cells. Previous studies have shown that certain Treg subsets are capable of producing pro-inflammatory cytokines such as IFN-γ, suggesting a more complex immunological role. Conversely, CD161 expression correlates negatively with less differentiated or immunologically “quiet” cells (M0 macrophages and naive CD4+ T cells), which do not themselves mount active immune attacks. In other words, CD161 high tumors tend to have fewer naive/undifferentiated immune cells and more activated effector T cells. This pattern, characterized by abundant effector and memory T cells alongside reduced infiltration of resting immune cells, is characteristic of an “immune-activated” tumor microenvironment. These findings are consistent with a recent pan-cancer analysis, which showed that tumors with high CD161 expression harbor increased cytotoxic and memory T cell infiltration and stronger interferon signaling (19).

Notably, CD161 expression was also strongly associated with several inhibitory checkpoint molecules (PD-1, BTLA, and TIGIT) as well as stimulatory immune checkpoints (such as CD27), suggesting a potential role in immune modulation. However, CD161 appears to mark a functionally active, “inflamed” T cell subset. Previous studies have shown that CD161 identifies a subset of cytotoxic T lymphocytes (CTLs) that co-express inhibitory receptors yet remain highly functional (18). These CD161^+^ CTLs are described as an inflamed, transitional population that responds effectively to checkpoint blockade (18). Moreover, co-expression of CD161 and PD-1 on T cells has been reported to promote IFN-γ release and PD-L1 upregulation in the tumor microenvironment (27). Such a feedback loop may enhance the efficacy of immune checkpoint inhibitors, further supporting CD161 as a predictive biomarker of favorable treatment response.

Our single-cell analysis further confirmed this phenomenon. Although CD161^+^ T cells express inhibitory checkpoint molecules such as PD-1 and CTLA-4, they also express markers including GZMB and SELL, indicating retained migratory capacity (SELL) and partial cytotoxic function (GZMB). Consequently, CD161-high T cells are likely suppressed by PD-1/CTLA-4 signaling but can restore their cytotoxic activity upon PD-1 blockade therapy. This may partially explain why high CD161 expression is associated with increased sensitivity to immune checkpoint inhibitors and improved therapeutic efficacy.

Consistent with these insights, our Kaplan–Meier curve analyses of both the GEO dataset and our immunotherapy-treated cohort demonstrated that high CD161 expression was significantly associated with prolonged PFS and OS in HNSCC patients receiving immunotherapy. Notably, although CD161 expression was evaluated at the transcriptomic level in the public datasets and at the protein level in our institutional cohort, both approaches yielded consistent associations with favorable clinical outcomes, underscoring the robustness of CD161 as a potential prognostic biomarker in HNSCC and supporting its role as a marker of antitumor immune activity and a promising guide for personalized immunotherapy strategies.

In conclusion, our findings suggest that CD161 may serve as a valuable biomarker for monitoring immunotherapy response in HNSCC and may represent a potential therapeutic target for enhancing treatment efficacy. Further research is needed to elucidate the underlying mechanisms and to explore CD161-directed strategies in combination with existing treatments.

Despite these promising results, this study has limitations. First, several analyses, including the immunohistochemical validation, were based on relatively small sample sizes. Therefore, large, multi-center cohorts will be required to validate the prognostic utility of CD161 in HNSCC. Second, although we observed significant correlations between CD161 expression and immune infiltration, experimental studies are needed to dissect the mechanistic role of CD161 in modulating tumor immunity. Third, although single-cell analysis suggested that CD161 was predominantly expressed in CD4^+^ T cell populations, the validation cohort evaluated overall CD161 protein expression by conventional IHC, and co-localization with CD4 or PD-1 was not assessed. Finally, the cohort included patients with different HNSCC subtypes, but was enriched for nasopharyngeal carcinoma; given its high prevalence and well-documented immunotherapy responsiveness, caution is warranted when generalizing these findings to all HNSCC subtypes.

Future studies incorporating multiplex immunofluorescence, spatial transcriptomics, and larger prospective cohorts are warranted to further characterize the cellular distribution and functional status of CD161-positive immune cells and to validate the association between CD161 expression and immunotherapy response in HNSCC. In addition, mechanistic investigations are needed to elucidate how CD161 shapes the tumor immune microenvironment, regulates T cell–mediated antitumor immunity, and contributes to immune evasion. Particular attention should be given to its interactions with other immune checkpoint pathways, including PD-1–related signaling. Such studies may provide important insights for the development of novel immunotherapeutic strategies. Ultimately, clinical trials evaluating CD161-targeted approaches or therapeutic modulation of CD161, particularly in combination with immune checkpoint inhibitors, may help establish its value as both a predictive biomarker and a therapeutic target. Given its fundamental role in immune regulation, CD161 may also have broader relevance across other malignancies characterized by immune escape.

## Data Availability

The datasets generated during and/or analyzed during the current study are available from the corresponding author on reasonable request.
