# Supplementary material for: CD161 serves as a prognostic biomarker and predicts immunotherapy response in head and neck squamous cell carcinoma
Source: Front Immunol. 2026 Aug 7;17:1855876. doi: 10.3389/fimmu.2026.1855876 (PMC13493243; doi:10.3389/fimmu.2026.1855876)
Supplement: Supplementary file 1 [file DataSheet1.doc]

Supplementary Figure 1. Volcano plot of differentially expressed genes comparing CD161-high and CD161-low subgroups stratified by CD161 median expression in the TCGA cohort.

|   A  D |   B |   C |
| --- | --- | --- |
|  |   E |   F |

Supplementary Figure 2. Distribution of CD161 expression in high- and low-expression groups stratified by optimal cutoff values derived from distinct survival endpoints across four independent cohorts. (A) TCGA-OS; (B) TCGA-DSS; (C) GSE102349-TTE; (D) GSE159067-PFS; (E) GSE159067-OS; (F) our institutional immunotherapy cohort. ***p < 0.001 (high vs. low expression groups).

Supplementary Figure 3. Differential expression of CD161 mRNA in various of cancers and corresponding normal tissues. *p < 0.05; **p < 0.01; ***p < 0.001.

Supplementary Figure 4. Cell from GSE103322 dataset were partitioned into 20 clusters using uniform manifold approximation and projection (UMAP).

Supplementary Figure 5. Marker genes expression in cell clusters.

Supplementary Figure 6.T cells were extracted and were divided into 5 clusters.

Supplementary Figure 7. Marker genes expression in different T cell clusters.
